# *TOR2A* Variants in Blepharospasm

**DOI:** 10.5334/tohm.825

**Published:** 2023-12-08

**Authors:** Samira Saeirad, Mark S. LeDoux

**Affiliations:** 1 University of Memphis, Memphis, Tennessee, USA; 2 Veracity Neuroscience LLC, Memphis, Tennessee, USA

**Keywords:** blepharospasm, *TOR2A*, Sanger sequencing, variant analysis, dystonia

## Abstract

**Background:**

Genetic factors have been implicated in the pathogenesis of blepharospasm (BSP), a dystonia characterized by excessive blinking and involuntary eyelid closure. Previous research identified a co-segregating deleterious *TOR2A* variant (GRCh38/hg38, NC_000009.12: g.127733410G>A, NM_001085347.3:c.568C>T, p. Arg190Cys) in three subjects with BSP and three carriers within a multi-generation pedigree. Other *TOR2A* variants have been reported in patients with dystonia.

**Methods:**

Sanger sequencing was used to screen a cohort of 307 subjects with isolated BSP or BSP-plus dystonia affecting additional anatomical segments (BSP+). We also utilized computational tools to uniformly assess the deleteriousness and potential pathogenicity of previously reported *TOR2A* variants.

**Results:**

There were no highly deleterious *TOR2A* variants in the coding or contiguous splice site regions of *TOR2A* within our cohort of 307 subjects.

**Discussion:**

Highly deleterious variants in *TOR2A* are rare in patients with BSP/BSP+ phenotypes.

**Highlights:**

Over 300 patients with BSP were screened for variants in *TOR2A*, a *TOR1A* (DYT1) homologue. No highly deleterious variants were identified in our cohort. The role of *TOR2A* in BSP and other forms of dystonia remains indeterminant.

## Introduction

Blepharospasm (BSP) is a focal dystonia, typically adult-onset, characterized by the occurrence of involuntary spasms of the orbicularis oculi muscles, which usually manifests bilaterally and demonstrates synchrony and symmetry [1]. Over time, BSP often extends to affect neighboring craniocervical regions, encompassing the lower face, masticatory muscles, and neck, leading to the development of segmental craniocervical dystonia [2,3]. The term “BSP-plus” (BSP+) is employed to describe individuals with BSP who exhibit further expansion of signs into these contiguous anatomical segments [4]. Many individuals with BSP have a first-degree relative with dystonia and penetrance is roughly 20% in BSP pedigrees [5,6]. BSP alone or in combination with dystonia in other anatomical segments has been reported in patients with deleterious variants in *TOR1A, THAP1* and *GNAL*. In biorepositories of isolated dystonia that include subjects with BSP, approximately 10% of participants report having a dystonia-affected relative [2,7,8].

*TOR2A*, encoding torsin2A, is a member of the AAA+ superfamily of ATPases and primarily associated with endoplasmic reticulum (ER) function [9]. The *TOR2A* homologue *TOR1A* was the first gene convincingly linked to isolated dystonia [10]. Mutations in *TOR2A* have been implicated in various dystonic phenotypes, including both isolated blepharospasm and more complex forms of dystonia [11,12,13]. In particular, a highly deleterious *TOR2A* variant was found to co-segregate in a large multiplex family with BSP (NM_001085347.3:c.568C>T, p. Arg190Cys) [11].

## Methods

The genetic material utilized in this investigation was sourced from specimens collected by the Dystonia Coalition (DC) and obtained from the Coriell Institute for Medical Research, located in Camden, New Jersey, USA. The DC operates as an integral component of the Rare Diseases Clinical Research Network, which receives funding support from the National Institutes of Health and is administered by the National Center for Advancing Translational Sciences (NCATS). This funding is provided through a collaborative grant (U54NS116025) involving NCATS and the National Institute of Neurological Disorders and Stroke. The ethical clearance for the DNA analyses conducted in this study was granted by the Institutional Review Board at the University of Memphis.

The cohort under investigation comprised 307 individuals with BSP phenotypes (N = 200) or BSP+ phenotypes (N = 107), which included a range of combinations involving lower facial, oromandibular, and cervical dystonia. The demographic breakdown included 224 females and 83 males, with ages spanning from 19 to 87 years. The median age at data acquisition was 63.1 ± 11.0 years. The racial composition consisted of 259 whites, 1 Native American, 1 Pacific Islander, 11 Asians, 20 blacks, 5 individuals with mixed racial backgrounds, and 10 individuals with undisclosed or unknown race.

For our genetic analyses, we employed the GRCh38.p14 reference genome assembly. Primers were designed to comprehensively cover *TOR2A* coding regions, including exon-intron boundaries (Table 1). Our sequencing efforts extended to proximal intergenic regions both 5’ and 3’ to *TOR2A*. We conducted unidirectional Sanger sequencing for the entire cohort, followed by bidirectional Sanger sequencing to validate the identified variants.

**Table 1:** *TOR2A* (GRCh38/hg38, NC_000009.12) primers for Sanger sequencing.


PRIMER NAME	SEQUENCE (5’ → 3’)	LOCUS	PRODUCT SIZE (bp)

*TOR2A*_5’UTR-F	CCT GAG CCT TCT TAC TGT GAA T	NC_000009.12: 127731790–769	306

*TOR2A*-5’UTR-R	GCC TCC TTC CAG AGC AAT TA	NC_000009.12: 127731505–485	

*TOR2A*-E1F	AGGAGCGTCGGGAGTTGTAG	NC_000009.12: 127735387–367	378

*TOR2A*-E1R	CTGGGTCCTCAGCTTCTCTG	NC_000009.12:127735032–010	

*TOR2A*-E2F	CCAGACCAGGTTCCAGACAT	NC_000009.12: 127734646–626	391

*TOR2A*-E2R	CACCCCATGGTGAGAACAG	NC_000009.12: 127734275–256	

*TOR2A*-E3F	TTGGGAAGAGGTCTGGTGTT	NC_000009.12: 127733627–607	300

*TOR2A*-E3R	AGCTGAACCTCTGAGAAGTGG	NC_000009.12: 127733349–328	

*TOR2A*-E4F	AGCGTTTTCAGTGGAGTTGG	NC_000009.12: 127732827–807	398

*TOR2A*-E4R	AGTCACAAAGCTGGGAGTGC	NC_000009.12: 127732847–827	

*TOR2A*-E5F	TCTCCCCTCTTGTGAAGCAC	NC_000009.12: 127732329–309	397

*TOR2A*-E5R	TCCGTTCATCTCACTTGGTG	NC_000009.12: 127731953–933	

*TOR2A*-3’UTR-F	AGC ACT AAT GGC ACA GAG TAA G	NC_000009.12: 127735703–681	345

*TOR2A*-3’UTR-R	GCC CAT CGC CTA CAA CTC	NC_000009.12: 127735377–359	


To identify previously reported *TOR2A* variants, we conducted systematic searches of ClinVar [14] and PubMed. PubMed searches were refined using specific search terms, including dystonia, blepharospasm, gene, genetics, mutation, genetic variant, Meige, and *TOR2A*. Additionally, we leveraged the gnomAD V3.1.2 database to gauge the population prevalence of these variants [15]. To assess the potential deleteriousness of variants, we utilized CADD Phred-scores [16,17], MetaLR [18,19], and REVEL [19]. Our classification of pathogenicity adhered to the established criteria of the American College of Medical Genetics and Genomics [20], which takes into account a variety of factors, including population data, variant databases, co-segregation, disease databases, and the variant’s location within established functional domains of the encoded protein. Variants were categorized using recommended terminology, including ‘pathogenic,’ ‘likely pathogenic,’ ‘uncertain significance,’ ‘likely benign,’ and ‘benign.’ Furthermore, we examined the gnomAD v3.1.2 dataset to identify putative loss-of-function (pLoF) variants.

## Results

No highly deleterious *TOR2A* variants were identified in our cohort of 307 subjects (Table 2). The major *TOR2A* isoform (NM_001085347.3, transcript variant 1) harbors 5 exons. Other RefSeq and Consensus Coding Sequence isoforms contain 2 to 4 exons. As seen in Table 2, several variants were identified but none of these showed notable differences in allele frequency when compared to the gnomAD v3.1.2 database. One variant (NC_000009.12:g.127733354C>G) is present in both coding (NM_130459.4:c.624G>C, p.Trp208Cys) and non-coding regions (NM_001085347.3:c.593+31G>C).

**Table 2:** *TOR2A* (GRCh38/hg38, NC_000009.12, NM_001085347.2) variants identified with Sanger sequencing.


VARIANT	NUMBER OF SUBJECTS	ALLELE FREQUENCY	HOMOZYGOTES	PROTEIN	GNOMAD V3.1.2 ALLELE FREQUENCY	CADDPHRED-SCALED	REVEL	MetaLR

NM_001085347.3:c.593+31G>C NM_130459.4:c.624G>C (rs564754)	234/307 (76.2%)	350/614 (57.0%)	58	NA p.Trp208Cys	97582/152038 (64.18%)	0.203	0.032	0

NM_001085347.3: c.721+32A>G (rs515182)	290/307 (94.5%)	370/614 (60.3%)	40	NA	101903/152022 (67.03%)	4.89	NA	NA

NM_001085347.3: c.607A>G (rs538066)	260/307 (84.7%)	560/614 (91.2%)	150	p. Lys203Glu	150579 /152218 (98.92%)	20.4	0.091	0

NM_001085347.3:c.793C>T (rs114990094)	10/307 (3.25%)	12/614 (1.95%)	0	p. Arg265Trp	3076/152220 (2.02%)	26.0	0.291	0.164


*TOR2A* is included in 62 ClinVar submissions. Of these, 40 are pathogenic structural variants affecting multiple genes. There are 17 missense variants of uncertain significance (Table 3) and several of these are deleterious and rare or absent from gnomAD v3.1.2.

**Table 3:** *TOR2A* variants reported by NCBI’s ClinVar and PubMed.


VARIANT(ACCESSION)	PROTEIN CHANGE	CONDITION (NUMBER OF PROBANDS)	CLINICAL SIGNIFICANCE	gnomAD V3.1.2 (ALLELE FREQUENCY)	CADDPHRED-SCALED	MetaLR	REVEL	REF.

c.194T>C (SCV003669886.1)	p. Leu65Pro	Inborn genetic disease (N = 1)	Uncertain significance	–	31.0	0.2864	0.664	[14]

c.247C>T (SCV003546031.1)	p. Pro83Ser	Inborn genetic disease (N = 1)	Uncertain significance	2/152200	28.3	0.2573	0.376	

c.338G>A (SCV003885945.1)	p. Gly113Asp	Inborn genetic disease (N = 1)	Uncertain significance	–	31.0	0.02109	0.024	

c.423T>A (SCV003950745.1)	p. Asp141Glu	Inborn genetic disease (N = 1)	Uncertain significance	2/152214	16.1	0.04569	0.031	

c.463C>T (SCV003757814.1)	p. Arg155Cys	Inborn genetic disease (N = 1)	Uncertain significance	2/152230	24.5	0.3835	0.281	

c.553T>C (SCV003661212.1)	p. Tyr185His	Inborn genetic disease (N = 1)	Uncertain significance	–	23.5	0.0976	0.182	

c.62T>C (SCV003534402.1)	p.Val21Ala	Inborn genetic disease (N = 1)	Uncertain significance	3/152194	14.8	0.1089	0.0540	

c.734A>G (SCV003566928.1)	p.Asn245Ser	Inborn genetic disease (N = 1)	Uncertain significance	1/231144	7.8	0.0521	0.040	

c.737C>T (SCV003615110.1)	p.Ser246Leu	Inborn genetic disease (N = 1)	Uncertain significance	2/152226	26.0	0.4321	0.563	

c.766G>A (SCV004004774.1)	p. Ala256Thr	Inborn genetic disease (N = 1)	Uncertain significance	6/276576	21.2	0.1399	0.089	

c.785C>G (SCV003708033.1)	p. Pro262Arg	Inborn genetic disease (N = 1)	Uncertain significance	4/152198	27.3	0.5009	0.809	

c.805C>T (SCV003951769.1)	p. Arg269Trp	Inborn genetic disease (N = 1)	Uncertain significance	7/279546	26.5	0.3993	0.172	

c.907C>A (SCV001041175.1)	p. Gln303Lys	Inborn genetic disease (N = 1)	Uncertain significance	67/152230	17.1	0.0676	0.146	

c.925G>A (SCV003551979.1)	p. Gly309Ser	Inborn genetic disease (N = 1)	Uncertain significance	9/152230	27.1	0.5849	0.813	

c.937G>A (SCV003529445.1)	p. Val313Met	Inborn genetic disease (N = 1)	Uncertain significance	6/152246	25.4	0.5539	0.658	

c.568C>T (NM_001085347.3)	p.Arg190Cys	BSP/BSP+ (N = 3, one pedigree)	Likely pathogenic	5/152188	29.2	0.548	0.5	[11]

c.593+31G>C (NM_001085347.2)	p. Trp208Cys	BSP	Benign	97582/ 152038	0.203	0.000	0.0320	[13]

c.-42G>A (NM_130459.3)	NA	BSP/BSP+ (N = 6)	Benign	12139/152166	12.2	–	–	[12]

c.277_288dup (NM_001085347.3)	p.Gly93_Gly96dup	BSP/BSP+ (N = 1 homozygote)	Uncertain significance	11/152226	21.5	–	–	

c.418–51T>G (NM_001085347.3)	NA	BSP/BSP+ (N = 35), Controls (N = 40)	Benign	98659/151956	10.3	–	–	

c.555C>T (NM_001085347.3)	p.Tyr185=	BSP/BSP+ (N = 1), Controls (N = 0)	Uncertain significance	9/152204	0.382	–	–	

c.593+36del (NM_001085347.3) c.629del (NM_130459.4)	NA p.Gly210AlafsTer60	BSP/BSP+ (N = 1), Controls (N = 0)	Uncertain significance	3/152204	0.81	–	–	

c.594–46C>T (NM_001085347.3)	NA	BSP/BSP+ (N=23 heterozygotes, 6 homozygotes), Controls (18 heterozygotes, 8 homozygotes)	Benign	18456/152198	0.252	–	–	

c.721+32A>G (NM_001085347.3)	NA	BSP/BSP+ (N=35 heterozygotes, 9 homozygotes), Controls (48 heterozygotes, 24 homozygotes)	Benign	101903/152022	4.89	–	–	

c.721+52G>A (NM_001085347.3)	NA	BSP/BSP+ (N = 10), Controls (N = 12)	Benign	25185/152088	7.55	–	–	

c.594–59C>T (NM_001085347.3)	NA	BSP/BSP+ (N = 1), Controls (N = 0)	Uncertain significance	7/152224	0.154	–	–	

c.721+83C>T (NM_001085347.3)	NA	BSP/BSP+ (N = 1), Controls (N = 0)	Uncertain significance	–	1.89	–	–	

c.594-55C>A (NM_001085347.3)	NA	BSP/BSP+ (N = 1), Controls (N = 0)	Uncertain significance	28/152228	6.55	–	–	

c.*28del (NM_001085347.3)	NA	BSP/BSP+ (N = 9), Controls (N = 8)	Benign	387/152220	0.128	–	–	

c.*125A>G (NM_001085347.3)	N/A	BSP/BSP+ (N = 1), Controls (N = 1)	Uncertain significance	1/152222	5.65	NA	NA	

c.786G>A (NM_001085347.3)	p.Pro262=	BSP/BSP+ (N = 1), Controls (N = 1)	Benign	83/152120	0.572	0.0614	0.0440	


Two independent studies screened patients with mainly BSP for *TOR2A* variants [12,13]. No highly deleterious (CADD > 25) variants were identified in their cohorts (Table 3). Co-segregation was not performed in either study and no family history information is provided for identified variants. A variant of undetermined significance was identified in a single subject with BSP (NM_001085347.3:c.593+36del; NM_130459.4:c.629del, p.Gly210AlafsTer60). This single nucleotide deletion results in a frameshift and likely nonsense mediated decay within transcript 2 (NP_569726.2:p.Gly210AlafsTer60). Another variant in the same manuscript appears to be incorrectly assigned to an intronic location (NM_001085347.3:c.289insGGCTGGACCGGC/c.299delC). The c.299C cannot be validated and is a likely annotation error due to misinterpretation of the electropherogram. The insertion is actually located in Exon 2 (NM_001085347.3:c.277_288dup, p.Gly93_Gly96dup) with a total allele count of 11/152226 and East Asian allele count of 10/5188 in gnomAD v3.1.2 and CADD-Phred score of 21.5.

## Discussion

Given our previous work showing co-segregation of a highly deleterious variant in a multiplex pedigree and the close similarity to *TOR1A*, we undertook a comprehensive analysis of *TOR2A* in BSP/BSP+. In our cohort of 307 subjects with BSP/BSP+, there were no highly deleterious *TOR2A* variants. Moreover, common single nucleotide polymorphisms showed no association with BSP/BSP+ when compared to a population cohort (gnomAD v3.1.2). Unified *in silico* analysis of two other screening studies of BSP identified several variants of uncertain significance.

Like *TOR1A, TOR2A* is located at 9q34.11. Also, like TorsinA, deletion of Torsin2A increases nuclear envelope blebbing [21]. Online Inheritance in Man does not yet link *TOR2A* to a human medical disorder. In gnomAD v3.1.2, there are a total of 11 unflagged putative loss-of-function (pLoF) coding variants. Numerous deleterious single nucleotide variants are reported in ClinVar but no trio analyses are included in the individual reports and most of these variants are present in normal populations. It is unlikely that these variants could cause severe early-onset phenotypes in Mendelian fashion but could contribute to oligogenic burden [22].

The previously published *TOR2A* variant (NM_130459.3:c.568C>T, p.Arg190Cys) variant [11] was not found in the DC cohort or two other screening studies [12,13] Importantly, this variant is predicted to be deleterious by various *in silico* tools and co-segregated with BSP in a four-generation pedigree. At the time of analysis, penetrance was 43% in this pedigree (3 affected, 4 carriers). Co-segregation was not assessed in the two other published screening studies.

The are several limitations to our work. Most importantly, we only examined patients with BSP/BSP+. It is possible that *TOR2A* plays a larger role in generalized and other anatomical distributions of dystonia. We only used Sanger sequencing which can fail to detect exonic deletions and larger structural variants. Our sample size was modest. For illustration, power analysis indicates that a sample size of 545 would provide 80% power (α = 0.05) to detect a pathogenic *TOR2A* allele in a BSP cohort if *TOR2A* contributes to 1% of BSP/BSP+ cases with a penetrance of 20%. Finally, we did not assess the biological effects of any variant with a read-out such as nuclear bleb formation.

In conclusion, highly deleterious variants in *TOR2A* are rare in BSP/BSP+ phenotypes. Future studies should include younger patients and other anatomical distributions of dystonia.
